# Stemness and clinical features in relation to the subventricular zone in diffuse lower-grade glioma: an exploratory study

**DOI:** 10.1093/noajnl/vdac074

**Published:** 2022-06-04

**Authors:** Alba Corell, Tomás Gómez Vecchio, Sandra Ferreyra Vega, Anna Dénes, Alice Neimantaite, Alexander Hagerius, Hanna Barchéus, Ole Solheim, Cecilia Lindskog, Thomas Olsson Bontell, Helena Carén, Asgeir S Jakola, Anja Smits

**Affiliations:** Department of Neurosurgery, Sahlgrenska University Hospital, Gothenburg, Sweden; Department of Clinical Neuroscience, Institute of Neuroscience and Physiology, The Sahlgrenska Academy, University of Gothenburg, Gothenburg, Sweden; Department of Clinical Neuroscience, Institute of Neuroscience and Physiology, The Sahlgrenska Academy, University of Gothenburg, Gothenburg, Sweden; Department of Clinical Neuroscience, Institute of Neuroscience and Physiology, The Sahlgrenska Academy, University of Gothenburg, Gothenburg, Sweden; Department of Clinical Neuroscience, Institute of Neuroscience and Physiology, The Sahlgrenska Academy, University of Gothenburg, Gothenburg, Sweden; Department of Clinical Neuroscience, Institute of Neuroscience and Physiology, The Sahlgrenska Academy, University of Gothenburg, Gothenburg, Sweden; Department of Clinical Neuroscience, Institute of Neuroscience and Physiology, The Sahlgrenska Academy, University of Gothenburg, Gothenburg, Sweden; Department of Clinical Neuroscience, Institute of Neuroscience and Physiology, The Sahlgrenska Academy, University of Gothenburg, Gothenburg, Sweden; Department of Neuromedicine and Movement Science, Norwegian University of Science and Technology, Trondheim, Norway; Department of Neurosurgery, St. Olavs University Hospital, Trondheim, Norway; Department of Immunology, Genetics and Pathology, Uppsala University, Uppsala, Sweden; Department of Clinical Pathology and Cytology, Sahlgrenska University Hospital, Gothenburg, Sweden; Department of Physiology, Institute of Neuroscience and Physiology, The Sahlgrenska Academy, University of Gothenburg, Gothenburg, Sweden; Sahlgrenska Center for Cancer Research, Department of Laboratory Medicine, Institute of Biomedicine, The Sahlgrenska Academy, University of Gothenburg, Gothenburg, Sweden; Department of Neurosurgery, Sahlgrenska University Hospital, Gothenburg, Sweden; Department of Clinical Neuroscience, Institute of Neuroscience and Physiology, The Sahlgrenska Academy, University of Gothenburg, Gothenburg, Sweden; Department of Neuromedicine and Movement Science, Norwegian University of Science and Technology, Trondheim, Norway; Department of Clinical Neuroscience, Institute of Neuroscience and Physiology, The Sahlgrenska Academy, University of Gothenburg, Gothenburg, Sweden; Department of Medicine, Neurology, Uppsala University, Uppsala, Sweden

**Keywords:** astrocytoma, methylation, oligodendroglioma, subventricular zone, tumorigenesis

## Abstract

**Background:**

The subventricular zone (SVZ) of the human brain is a site of adult stem cell proliferation and a microenvironment for neural stem cells (NSCs). It has been suggested that NSCs in the SVZ are potential cells of origin containing driver mutations of glioblastoma, but their role in the origin of diffuse lower-grade gliomas (dLGGs) is not much studied.

**Methods:**

We included 188 patients ≥18 years with *IDH*-mutated dLGG (WHO grades 2–3) histologically diagnosed between 2007 and 2020. Tissue microarrays of tumor samples for patients between 2007 and 2016 were used for immunodetection of Nestin, SOX2, SOX9, KLF4, NANOG, CD133 cMYC, and Ki67. DNA methylation profile was used for stemness index (mDNAsi). Tumor contact with the SVZ was assessed and the distance was computed.

**Results:**

Overall, 70.2% of the dLGG had SVZ contact. Tumors with SVZ contact were larger (102.4 vs 30.9 mL, *P* < .01), the patients were older (44.3 vs 40.4 years, *P* = .04) and more often had symptoms related to increased intracranial pressure (31.8% vs 7.1%, *P* < .01). The expression of SOX2, SOX9, Nestin, and Ki67 showed intersample variability, but no difference was found between tumors with or without SVZ contact, nor with the actual distance to the SVZ. mDNAsi was similar between groups (*P* = .42).

**Conclusions:**

We found no statistical relationship between proximity with the SVZ and mDNAsi or expression of SOX2, SOX9, Nestin, and Ki67 in *IDH*-mutated dLGG. Our data suggest that the potential impact of SVZ on *IDH*-mutated dLGG is probably not associated with a more stemness-like tumor profile.

**Key Points**

- There was no relation between SVZ and SOX2, SOX9, Ki67, and Nestin in *IDH*-mutated dLGG.
- Methylation-based stemness index was alike between dLGG with or without SVZ contact.

**Importance of the Study**

The subventricular zone (SVZ) of the brain is a source of adult stem cells and harbors a specialized microenvironment that facilitates the survival and regulation of neural stem cells. This area has been studied in relation to glioblastomas, but not as much studied in diffuse lower-grade gliomas (dLGGs). There was no statistical relationship between SVZ and expression of SOX2, SOX9, Ki67, and Nestin in *IDH-*mutated dLGG. Furthermore, the DNA methylation-based stemness index mDNAsi showed no significant difference between dLGG with or without SVZ contact.

Diffuse lower-grade gliomas (dLGGs) are slow growing primary brain tumors characterized by the presence of isocitrate dehydrogenase gene (*IDH*) mutation. dLGG occurs as either astrocytomas or oligodendrogliomas, where the latter have loss of chromosomal arms 1p and 19q.^1–3^ The hypothesis of gliomas originating from differentiated astrocytes and their precursor cells has been challenged by the discovery of neural stem cells (NSCs).^4–6^ It is now believed that gliomas can emerge from mutations in NSCs, but the role of NSCs in the origin of dLGG is still unclear.^5,7^

The subventricular zone (SVZ) is a source of adult stem cells and harbors a specialized microenvironment that facilitates the survival and regulation of NSCs.^6,8^ However, the exact cellular and molecular mechanisms behind this specialized microenvironment are not understood. The SVZ can be expected throughout the entire lateral ventricular lining, as it resides along the lateral wall of the lateral ventricles, with varying degree of thickness and cell densities.^9–11^ There are at least 4 types of cells in the SVZ, of which astrocytes may act as NSCs in a specialized SVZ niche.^12,13^ The organization of the SVZ is described as a composition of 4 layers: (1) the ependymal layer, (2) the hypocellular gap, (3) the astrocytic ribbon, and (4) the transitional zone to the parenchyma, which is rich in myelin and oligodendrocytes.^7^

The plasticity of cells in the SVZ not only comes with benefits for the adult brain (eg, tissue regeneration). It has been shown that NSCs in the SVZ possess remarkable similarities to brain tumor stem cells (BTSCs), where NSCs and BTSCs share multipotent and migratory capabilities.^7^ One hypothesis is that NSCs transform into BTSCs that migrate from the SVZ to other regions of the brain where they participate in tumorigenesis.^7,14^ It has been demonstrated that NSC in the SVZ secrete factors acting as chemoattractants, suggesting that tumor invasion of the SVZ may be facilitated by the SVZ niche.^15^ In line with this, several studies have used proximity to the SVZ as a marker of tumor origin.^13,16^

The term stemness, originally attributed to normal stem cells, is defined as the potential for self-renewal and differentiation of the cell of origin.^17^ As cancer cells progress, the tumor acquires stem-cell-like features with multitask capabilities in the initiation, maintenance, and progression of cancer.^18^ Cancer stemness has been widely investigated and is characterized by specific genomic, epigenomic, transcriptomic, and proteomic signatures. As such, the stemness methylation-based index (mDNAsi) reflects features of cancer stemness, while mRNA expression-based stemness index (mRNAsi) gives an indication of cancer stemness-related gene expression.^19^ In gliomas, mDNAsi shows a strong association with World Health Organization (WHO) malignancy grade and molecular subtypes, that is high mDNAsi correlating with more aggressive gliomas as well as specific molecular subtypes.^20^ Since the SVZ is a stem cell niche and a possible site of origin for *IDH-*mutated (*IDH*-mut) gliomas, it is tempting to speculate that glioma stemness is affected by proximity to SVZ.^21^ However, some studies also support a common path of origin of gliomas.^22^

Although the exact link between cancer stem cell markers and normal stem cells remains controversial, the expression of pluripotent stem cell markers such a CD133, NANOG, SOX2, and KLF4 is of interest to study in glioma.^23^ The stem cell markers selected for this study have all been identified in previous dLGG studies and attributed a putative role as prognostic biomarker in these tumors.^4,24–34^ Thus, these markers could be used for prognostication in clinical cohorts.^35^ In a longer perspective, some of these markers may also act as a possible biological targets, using antibody-based therapies specific for the different targets.^36^ Here, we have used a cohort of *IDH*-mutated dLGG for analysis of stem cell marker expression and DNA methylation-based stemness index to investigate the association between tumor stemness and its proximity to the SVZ, as well as to clinical characteristics. Our hypothesis is that *IDH-*mut gliomas in contact with SVZ show differential expression of stem cell markers compared to *IDH-*mut gliomas without SVZ contact, representing different underlying biology.

## Patients and Methods

### Patient Cohort

We identified patients ≥18 years with supratentorial dLGG (WHO grade 2 or 3 *IDH*-mut gliomas, according to WHO classification of tumors in central nervous system,^1,37^ treated surgically and histopathologically diagnosed at the Sahlgrenska University Hospital, Gothenburg, Sweden between 2007 and 2020 (*n* = 188). Data were obtained through electronic health records, pathology database, and operation logs. Tumors that were previously classified according to WHO 2007 were reclassified according to the WHO 2016 criteria for the purpose of the study. Reclassification according to the WHO 2016 CNS classification system, based on *IDH* mutation and 1p/19q codeletion status, was performed as previously described by Ferreyra Vega et al.^38^ The new classification system from 2021 did not change the nomenclature further.^39^

Of the 188 included patients in this study, 171 patients had available magnetic resonance imaging (MRI) for measurement of distance to SVZ (see *Tumor distance to the SVZ*) and 121 tumor samples were available for tissue microarray (TMA) analysis (see *Tissue handling and TMA construction*).

### Clinical and Baseline Radiological Variables

The following clinical variables were assessed: patient age, gender, symptoms at presentation, type of surgery, and histopathological diagnosis. Radiological variables included contact with SVZ, tumor location in relation to the SVZ, and preoperative tumor volume. Radiological and molecular assessments are described in the following sections.

### Involvement of the SVZ

Involvement of the SVZ was defined as contact between the tumor region and the subventricular area. If the tumor was in contact with the SVZ, further subclassification of the lateral ventricles was performed into the 4 regions of either anterior horn, body, atrium/occipital horn, or temporal horn, as previously described by Rhoton.^40^ We used preoperative MRI with fluid-attenuated inversion recovery (FLAIR) sequences, or T2-weighted images when FLAIR images were unavailable, in a dichotomous fashion (tumor in contact with SVZ, yes or no). A hyperintensive signal was considered tumor, since tumor edema in dLGG is rare.^41^ Assessment of tumor relation to the SVZ was performed by 2 trained investigators (A.H. and A.C.) under supervision of a senior neurosurgeon (A.S.J.) with considerable experience with dLGG imaging. Difficult cases with regard to assessing the involvement with SVZ were discussed with A.S.J. and a consensus-based decision was made thereafter. The preoperative tumor volume was segmented in a semi-manual fashion according to a previously described method^42^ using the segmentation software 3D slicer.^43^ There were multiple trained personnel performing segmentations of tumors being blinded to the clinical status of the subjects, but all segmentations were verified by a neurosurgeon (A.S.J.).

### Tumor Distance to the SVZ

**Figure 1.**
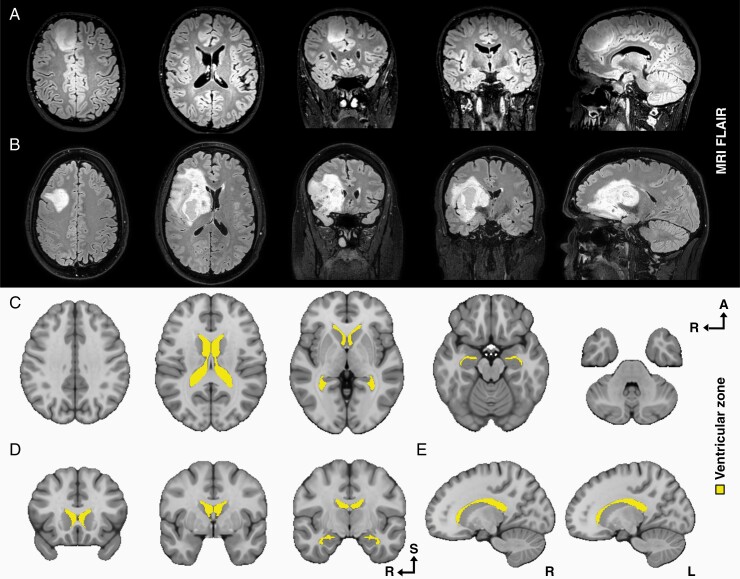
The images show examples of the qualitative assessment for the involvement of the subventricular zone in preoperative magnetic resonance imaging with fluid-attenuated inversion recovery and the anatomical definition of the ventricular zone used as target for contact and distance measurements in MNI space. (A) Axial, coronal, and sagittal slices of a tumor not in contact with the ventricular zone; (B) axial, coronal, and sagittal slices of a tumor in contact with the ventricular zone; (C) target for contact in axial slices (36, 18, 0, −18, −36); (D) target for contact in coronal slices (20, 0, −10); and (E) target for contact in sagittal slices (15(R) and −15(L)). MNI, Montreal Neurological Institute.

Tumor distance from the centroid of the tumor to the closest SVZ was calculated using tumor annotations on available preoperative MRIs (*n* = 171). The outline of the SVZ was annotated in the image used as registration target, by segmentation of the anatomical ventricles (see Figure 1).

Standard preprocessing was done using Functional Magnetic Resonance Imaging of the Brain Software Library (FLS) as described previously.^44^ MRIs were individually registered to the Montreal Neurological Institute (MNI) space, of which the T1 symmetric MNI 09a was used as registration target.^45^ Tumor segmentations and MRIs transformed to MNI space were individually controlled for errors or unexpected deformations by raters with experience in glioma image analysis (A.C., A.N., and T.G.V.). In a few cases (*n* = 11) registration was modified by adjustment of registration parameters.

For computation of tumor distance to SVZ, the shortest distance between the segmented tumor centroid point and the closest border of the outlined SVZ was measured. In multifocal lesions, the largest lesion was considered for centroid point calculation. Annotation centroid and distance calculation were done using Python programming language version 3.8.3 (Python Software Foundation).

### Tissue Handling and TMA Construction

A total of 121 tumor samples were available for TMA construction. Tumor tissue was fixed with 4% formaldehyde, dehydrated and embedded in paraffin wax, as previously described.^46^ A clinical histopathology workflow was performed for each sample based on routine stainings, immunohistochemistry, and next generation sequencing. In the next step, paraffin blocks of dLGG were used for production of TMAs. TMAs were generated as previously described, including 2 tissue cores from each donor block.^47^

### Immunohistochemical Staining of Stem Cell Markers and Ki67

Immunohistochemical staining of TMA slides were performed by the Human Protein Atlas facilities Tissue profiling site, at the Department of Immunology, Genetics and Pathology, Rudbeck Laboratory, Uppsala University, Sweden as previously described.^47^

The following antibodies were used for semiquantitative analysis of protein expression: anti-CD133 (1:50, HPA004922, Atlas Antibodies, Bromma), anti-Nestin (1:2500, HPA026111, Atlas Antibodies, Bromma), anti-SOX2 (1:50, 371R-15, Cell Marque), anti-SOX9 (1:1500, AMAb90795, Atlas Antibodies, Bromma), anti-Ki67 (1:800, M7240, Agilent), anti-NANOG (1:400, 2929.00.02, OriGene Technologies, Inc.), anti-KLF4 (1:100, HPA002926, Atlas Antibodies, Bromma), and anti-cMYC (1:150, sc-789, Santa Cruz Biotechnology, Inc.). Two of 3 observers (A.D., H.B., and A.S.) independently evaluated the staining results. An assessment of the proportion of immunoreactive tumor cells in each sample, as previously described.^48^ For this analysis, the entire piece of microtissue was examined. Results were then reviewed by a neuropathologist (T.O.B.).

**Figure 2.**
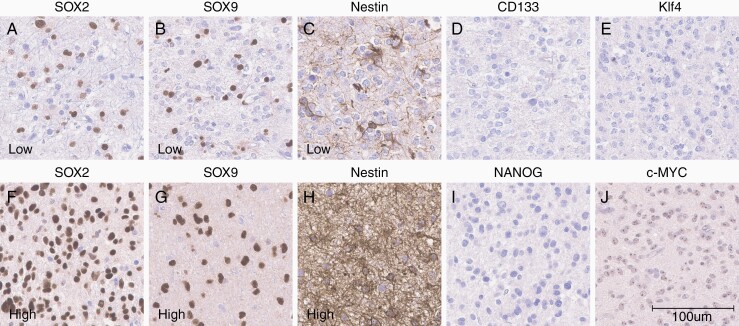
Immunohistochemical staining of SOX2 (A and F), SOX9 (B and G), and Nestin (C and H). Examples of staining patterns with low proportion of positive cells are shown in panels A, B, and C, while examples of staining patterns with high proportion of positive cells are shown in the panels F, G, and H. CD 133, Klf4, and NANOG showed negative immunostaining in all samples (D, E, and I, respectively). Immunohistochemical staining of c-MYC showed nucleolar staining pattern in all samples (panel J).

**Figure 3.**
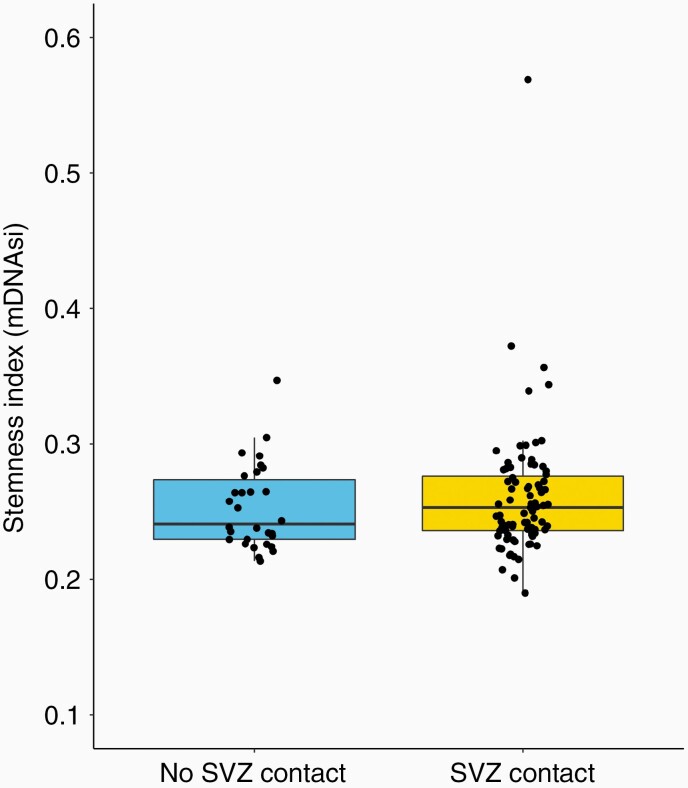
Overview of the distribution of mDNAsi in the 113 dLGG samples used in the methylation assay, tumors without contact with SVZ represented on the left side and tumors with SVZ contact represented on the right side.

Markers with intersample variability in expression (SOX2, SOX9, Nestin, and Ki67) (Figures 2 and 3) were included in further statistical analysis, while markers with uniformly negative (KLF4, NANOG, and CD133) or positive (cMYC) expression in all samples (Figure 2) were excluded. In a final step, immunoreactivity patterns of SOX2 (high proportion of positive cells) and NANOG (negative staining) in human dLGG were validated and confirmed by external collaborative party (Atlas Antibodies, Bromma).

Slides of stained TMA sections were scanned in 40× magnification using Aperio AT2 (Leica Biosystems) automated scanning system and assessed in the Aperio ImageScope software (Leica Biosystems). The digital images for each antibody were annotated manually with regard to the proportion of immunopositive cells, as well as the subcellular localization of immunostaining (nuclear, cytoplasmic, and membranous). Cutoffs for statistical analysis were determined before exploring, with the intention to create balanced group sizes, see Supplementary Figure 1 and Supplementary Table 1.

### DNA Methylation Analysis, the Stemness Methylation-Based Index mDNAsi

DNA was isolated from the paraffin blocks for all dLGG with available tissue for analysis (*n* = 117) and DNA methylation data were generated using with the Illumina MethylationEPIC array as previously described.^38^ The methylation-based stemness index (mDNAsi) was predicted according to Malta et al.^20^

### Statistical Analysis

DNA methylation data analysis was performed using the statistical software R with Rstudio (version 4.0.2). All remaining analysis was performed were performed with the SPSS, ver. 24.0 software (SPSS Inc.). Statistical significance level was set to *P* < .05 and all tests were 2-sided. Central tendencies were presented as means ± SD, or median and first quartile (Q1) and third quartile (Q3) if skewed. Categorical data were analyzed with Pearson’s chi-square test, but in 2 × 2 tables we used Fisher’s exact test due to small sample size. For ordinal data, Mann–Whitney *U*-test was used for analysis when appropriate. For continuous data, independent sample *t*-test was used or Mann–Whitney *U*-test, as appropriate depending on normal distribution as assessed from *Q*–*Q* plots. For analysis of distance from centroid of tumor to the SVZ simple linear regression was performed, in addition to multivariable linear regression using distance from tumor centroid as response. Age, sex, and percentage of positive core cells of SOX2, SOX9, Nestin, and Ki67 were used as dependent variables.

### Ethics Statement

This project was approved by the Regional ethical review board in Gothenburg, Sweden (DNR 1067-16 and DNR 363-17).

## Results

### Clinical and Radiological Characteristics

In the studied cohort (*n* = 188), 132 patients (70.2%) had an *IDH*-mut dLGG in contact with the SVZ. The patients with tumors in contact with SVZ were older (44.3 years) compared to those without SVZ contact (40.4 years; *P* = .04). As shown in Table 1, the presenting symptoms differed between patients with dLGG *IDH*-mut with and without contact with the SVZ; patients with tumors in contact with the SVZ more frequently presented with symptoms related to increased intracranial pressure, such as headache, nausea, and vomiting (*P* < .01).

**Table 1.** Clinical characteristics and radiological features, *IDH*-mut dLGG (*n* = 188)

	SVZ contact (*n* = 132)	No SVZ contact (*n* = 56)	*P*
Age, mean (SD)	44.3 (13.32)	40.4 (13.2)	.04
Female (%)	60 (45.5)	19 (54.5)	.15
Symptom at diagnosis^a^			
Asymptomatic	6 (4.5)	7 (12.5)	.06
Seizure	92 (69.7)	43 (76.8)	.38
Related to increased intracranial pressure	42 (31.8)	4 (7.1)	<.01
Motor deficit	13 (9.8)	1 (1.8)	.07
Language deficit	12 (9.1)	2 (3.6)	.24
Visual deficit	7 (5.3)	1 (1.8)	.44
Cognitive changes	19 (14.4)	5 (8.9)	.35
Other symptoms	21 (15.9)	7 (12.5)	.66
Main lobe involved^b^			
Frontal	75 (56.8)	40 (71.4)	.72
Temporal	33 (25.0)	4 (7.1)	<.01
Location dLGG in SVZ			
Frontal horn	60 (45.5)	0	<.01
Body of ventricle	28 (21.2)	0	<.01
Occipital horn	19 (14.4)	0	<.01
Temporal horn	25 (18.9)	0	<.01
No contact	0 (0.0)	56 (100.0)	<.01
Size			
Preoperative tumor volume, mean mL (SD)^c^	102.4 (76.3)	30.9 (23.2)	<.01
Type of surgery			
Resection	124 (93.9)	56 (100.0)	.11
1p/19q codeletion, *n* (%)	58 (43.9)	30 (53.6)	.26
WHO grade 2, *n* (%)	64 (48.5)	33 (58.9)	.21
WHO grade 3, *n* (%)	68 (51.5)	23 (41.1)	.21

dLGG, diffuse lower-grade glioma; SVZ, subventricular zone.

^a^More than 1 symptom at presentation possible. Other symptoms included paresthesia, vertigo, dysphagia, among others.

^b^Missing data for 3 patients.

^c^Missing data for 24 patients.

The frontal lobe was the most common tumor location, irrespective of SVZ contact (*P* = .72). As for the tumors in contact with SVZ, the frontal horn was the most common location (45.5%) followed by body of ventricle (21.2%). Furthermore, tumors in contact with the SVZ were larger mean volume compared to the no contact group, 102.4 vs 30.9 mL (*P* < .01). 43.9% of the gliomas in the SVZ contact group were oligodendrogliomas (ie, *IDH*-mut gliomas with 1p/19q codeletion) compared to 53.6% in the no contact group (*P* = .26).

### Molecular Characteristics of Tumor Samples

TMA was available for 121 tumors. The distribution of stem cell markers and the proliferation marker Ki67 in relation to the proximity of the tumor to the SVZ are shown in Table 2. The TMA analysis was performed according to methods stated under Patients and methods, *Tissue handling and TMA construction* and *Immunohistochemical staining of stem cell markers and Ki67.* Categorization of the fraction positive cells for each antibody into different scales is shown in Supplementary Table 1. The markers cMYC, showing equally positive expression in all samples, and KLF4, NANOG, and CD133 that were uniformly negative, were not further analyzed. No statistically significant differences in expression of SOX2, SOX9, Nestin, or Ki67 were found between patients with tumors with or without SVZ contact, see Table 2. Stemness index based on DNA methylation was similar in the 2 groups, 0.26 in the SVZ contact group and 0.25 in the SVZ no contact group (*P* = .42). See Figure 3 for an overview of the distribution of mDNAsi in the 113 samples used in the methylation assay.

**Table 2.** Results from immunostainings and DNA methylation stemness analysis in relation to SVZ proximity in *IDH*-mut dLGG WHO grades 2 and 3 (*n* = 121)

	SVZ contact (*n* = 91)	No SVZ contact (*n* = 30)	*P*
SOX2, *n* (%)		*n* = 29	.46
<70%	12 (13.2)	6 (20.7)	
70%–90%	34 (37.4)	12 (41.4)	
≥70%	45 (49.5)	11 (37.9)	
SOX9, *n* (%)		*n* = 29	.23
<50%	18 (19.8)	4 (13.8)	
50%–70%	30 (33.0)	6 (20.7)	
≥70%	43 (47.3)	19 (65.5)	
Nestin, *n* (%)			.50
≤10%	13 (14.3)	3 (10.0)	
10%–50%	57 (62.6)	17 (56.7)	
≥50%–90%	21 (23.1)	10 (33.3)	
Ki67, *n* (%)	*n* = 90		.98
<1%	34 (37.8)	12 (40.0)	
1%–4%	40 (44.4)	13 (43.3)	
≥4%	16 (17.8)	5 (16.7)	
Stemness index	*N* = 83		
Methylation stemness index, mean (SD)	0.26 (0.048)	0.25 (0.031)	.42

dLGG, diffuse lower-grade glioma; SVZ, subventricular zone.

### Distance From Centroid of Tumor to SVZ

**Figure 4.**
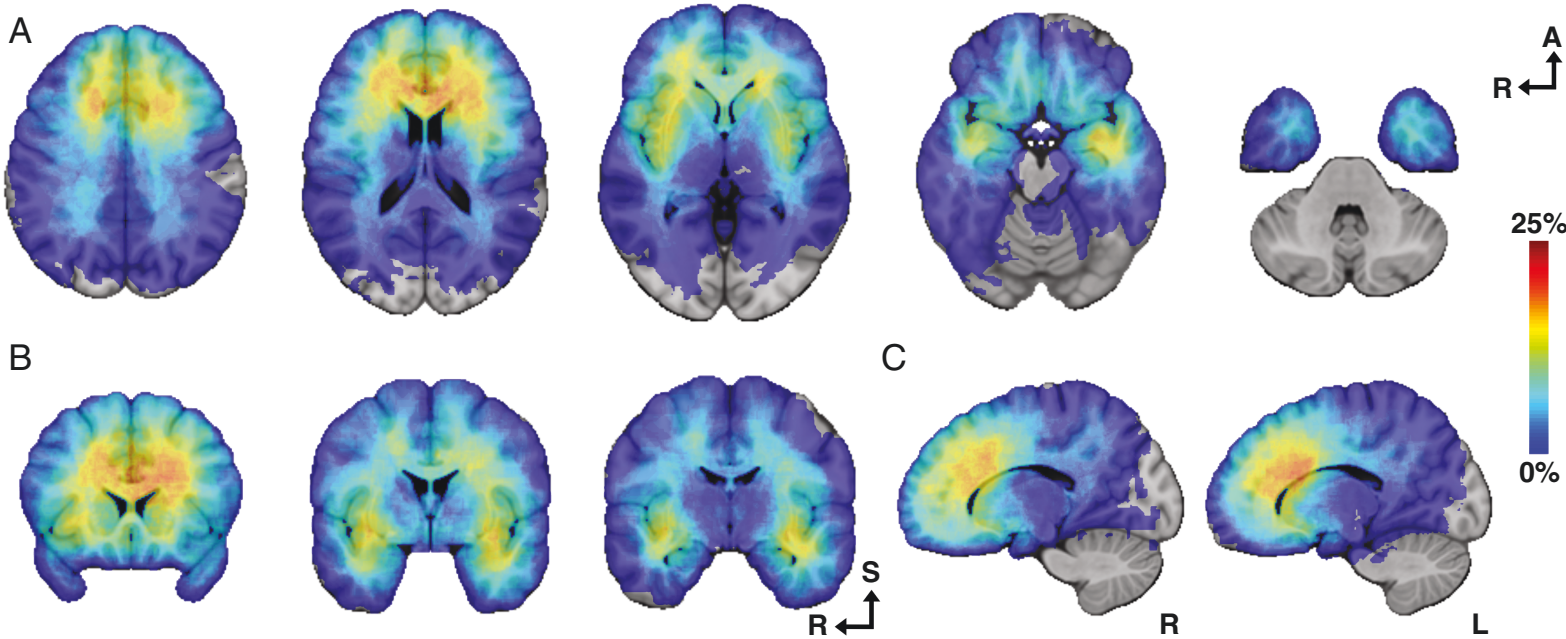
Gradient maps reconstructed from the fusion of each of the 171 tumor regions within the Montreal Neurological Institute (MNI) space. (A) Coordinates for axial slices: 36, 18, 0, −18, −36. (B) Coordinates for coronal slices: 20, 0, −10. (C) Coordinates for sagittal slices: 15(R), −15(L). The color intensity represents the voxel-based perceptual distribution of the tumor segmentations.

Tumor distance to SVZ was calculated for 171 patients. Exclusion was due to missing data (*n* = 7) and in those cases where registration was not feasible (*n* = 10). See Figure 4 for an overview of tumor locations in MNI space. The mean distance from the SVZ to the centroid of tumor was 24.0 mm (SD 9.0).

### Distance From Centroid of Tumor to SVZ in Relation to Stem Cell Markers and Stemness

To further analyze the correlation between expression of cell markers (SOX2, SOX9, Nestin, and Ki67) and the proximity to the SVZ, we used the distance from centroid of tumor to SVZ. In simple linear regression models, we investigated the possible relationship between the distance from the centroid of the tumor to the SVZ and the molecular characteristics of the tumor. There were no significant correlations between *IDH*-mut dLGG in proximity with the SVZ and the expression of SOX2 (*P* = .644), SOX9 (*P* = .882), Nestin (*P* = .908), or Ki67 (*P* = .614). Furthermore, linear regression analysis showed no correlation between methylation stemness index and distance from the centroid of tumor to the SVZ (*P* = .33).

Multivariable linear regression was performed to ascertain the possible relation between distance from tumor centroid to SVZ and age, sex, and percentage of positive core cells of SOX2, SOX9, Nestin, and Ki67. None of these variables were found significantly expressed in relation to distance to the SVZ: SOX2 (*P* = .545), SOX9 (*P* = .959), Nestin (*P* = .490), or Ki67 (*P* = .598).

## Discussion

We studied a cohort of patients with *IDH*-mut dLGG, and examined stem cell markers in relation to the SVZ. There were no statistically significant correlations for any of the selected stem cell markers with proximity to the SVZ. Neither did we find a correlation between the stemness index of the tumor and the proximity to the SVZ. Thus, the stemness phenotype in *IDH*-mut dLGG does not seem to be linked to the location of the tumor in relation to the SVZ.

From the clinical characteristics, it was apparent that larger tumors more often reached the SVZ. It is clear that the contact with the SVZ is directly related to the size of the tumor. Size also influences the clinical manifestation, since patients with tumors in proximity with the SVZ commonly had symptoms related to significant mass effect. An intriguing, as well as a logical finding was that larger tumor size was associated with higher age in this homogeneous cohort of *IDH-*mut dLGG WHO grades 2 and 3. This speaks in favor of the proposed idea that these tumors—in average—arise around the same chronological age.^49^

We used 2 measures for classification of SVZ contact; a dichotomous method according to Rhoton’s classification of the SVZ,^40^ and measurement from the centroid of the tumor as performed by Steed et al.^50^ Since none of the stem cell markers or the proliferation marker Ki67 were associated with the distance to the SVZ, we cannot draw any other conclusion from our analysis than that tumor size rather than tumor phenotype affects the likelihood of being in contact with the SVZ. The discrepancy between these results and previous findings is probably related to the more homogeneous group of tumors we used, illustrating the importance of tumor stratification analyzing only tumors with similar molecular characteristics.^48^ Future studies based on guided biopsies derived from lesions with and without SVZ contact, could possibly bring further clarification on this matter.

Similarly, we found that the stemness index, based on DNA methylation profiles, was not associated with proximity of the tumor to the SVZ. Previous reports of stemness index have included gliomas of mixed grades and different *IDH-*mutational status, which may explain the findings in our data.^20^ Interestingly, studies investigating the origin of low-grade glioma have suggested a different stem cell origin of *IDH*-wild type and *IDH*-mutant gliomas, with the former more often located to the subgranular zone in the hippocampus and the latter more often to the SVZ.^21^ Even though a higher expression of stem cell markers or higher stemness index in tumors with proximity to the SVZ could not be observed, it is important to realize that our study does not shed light on the site of origin and thus does not exclude a different origin according to tumor location. For instance, Lee et al. investigated NSC of the SVZ in glioblastoma mouse models and discovered that NSC similar to astrocytes can carry mutations from the SVZ to more distant regions of the brain and cause glioblastoma.^16^ It is possible that the SVZ provides a beneficial stem niche also for tumors in other ways that are not reflected by the tumor stemness itself. Also, due to unknown factors related to cell migration, theoretically neoplastic origin may still be BTSC in the SVZ even if tumors are located far away.

When studying tumors in relation to any specific area of the brain, the potential bias of tumor size has to be considered. Larger tumors are more likely to get into contact to any region. The distance from the centroid of tumor is therefore probably a better indication of the proximity to the SVZ, and also reflects the postulated oldest part of the tumor.^51^ Using this parameter in our analyses, we still did not find any statistically significant association between stem cell markers or stemness with proximity to the SVZ.

### Strengths and Limitations

One strength of the study is that only patients with *IDH*-mut dLGGs were analyzed, without no further stratification, thus representing external validity with data from a population-based setting is considered to be satisfactory. Another strength of this study is that the distance to the SVZ was obtained by both a dichotomous variable and a continuous measurement from the centroid of tumor, making also statistical analysis with continuous variable possible. Limitations include the retrospective data design and a bias in the acquisition of tumor samples for TMA, where a certain amount of tumor tissue is needed for the analysis, favoring tumor resections over biopsies. It should also be kept in mind that the stemness profile of the tumor, in term of the expression of stem cell markers, cannot be regarded as a biologically steady state. Migration of glioma stem cells into the SVZ has previously been suggested as an escape route for cells that reside in the SVZ. These cells, which do not originate from the SVZ themselves, may contribute to later tumor recurrences.^13^ Furthermore, the expression of stem cell markers in gliomas could change over time due to cellular plasticity and thereby differ from their cancer cell of origin.^52^

## Conclusion

We found no statistical relationship between proximity with the SVZ and stemness index or with expression of SOX2, SOX9, Ki67, and Nestin supporting our hypothesis that tumors in proximity to the SVZ have a more stemness-like phenotype in *IDH*-mutated dLGG. However, dLGG with SVZ contact was larger and these patients presented more often with symptoms from increased intracranial pressure. Our data suggest that the potential impact of SVZ on *IDH*-mutated dLGG is probably not associated with a more stemness-like profile of the tumor.

## Supplementary Material

vdac074_suppl_Supplementary_Material

## Notes

**Authorship Statement**. A.C., A.S., and A.S.J. planned the study design. A.C., A.H., H.B., A.N., A.D., S.F.V., and T.G.V. performed the clinical and radiological data collection. The immunohistochemical analysis was performed by A.D., T.O.B., and A.S. A.D. and H.B. performed the annotation of immunohistochemical staining under supervision of T.O.B. A.C. and A.H. performed the tumor volume segmentation. S.F.V. and H.C. performed the methylation analysis regarding stemness index. A.C., A.D., S.F.V., T.G.V., and A.S. wrote the manuscript and were involved in the submission process. A.C., T.G.V., S.F.V., A.D., A.N., A.H., H.B., O.S., C.L., T.O.B., H.C., A.S.J., and A.S. revised the manuscript. A.S. supervised the manuscript writing process. All authors substantively revised the manuscript and have approved the submitted version.
