# Supplementary material for: Stemness and clinical features in relation to the subventricular zone in diffuse lower-grade glioma: an exploratory study
Source: Neurooncol Adv. 2022 Jun 4;4(1):vdac074. doi: 10.1093/noajnl/vdac074 (PMC9248775; doi:10.1093/noajnl/vdac074)

**Supplementary Tables**

**Supplementary Table 1.** Categorization of positive cells for each marker.

|  | **Category** | | |
| --- | --- | --- | --- |
| **Marker** | **1** | **2** | **3** |
| SOX2, % | <70% | ≥70% – <90% | ≥90% |
| SOX9, % | <50% | ≥50% – <70% | ≥70% |
| Nestin, % | <10% | ≥10% – <50% | ≥50% |
| Ki67, % | <1% | ≥1% – <4% | ≥4% |

**Supplementary Figures**

**Supplementary Figure 1.** Diagram over distribution of dLGG samples in different staining categories for stem cell markers SOX2 (burgundy), SOX9 (green) and Nestin (blue) in diffuse lower-grade glioma (dLGG) samples.


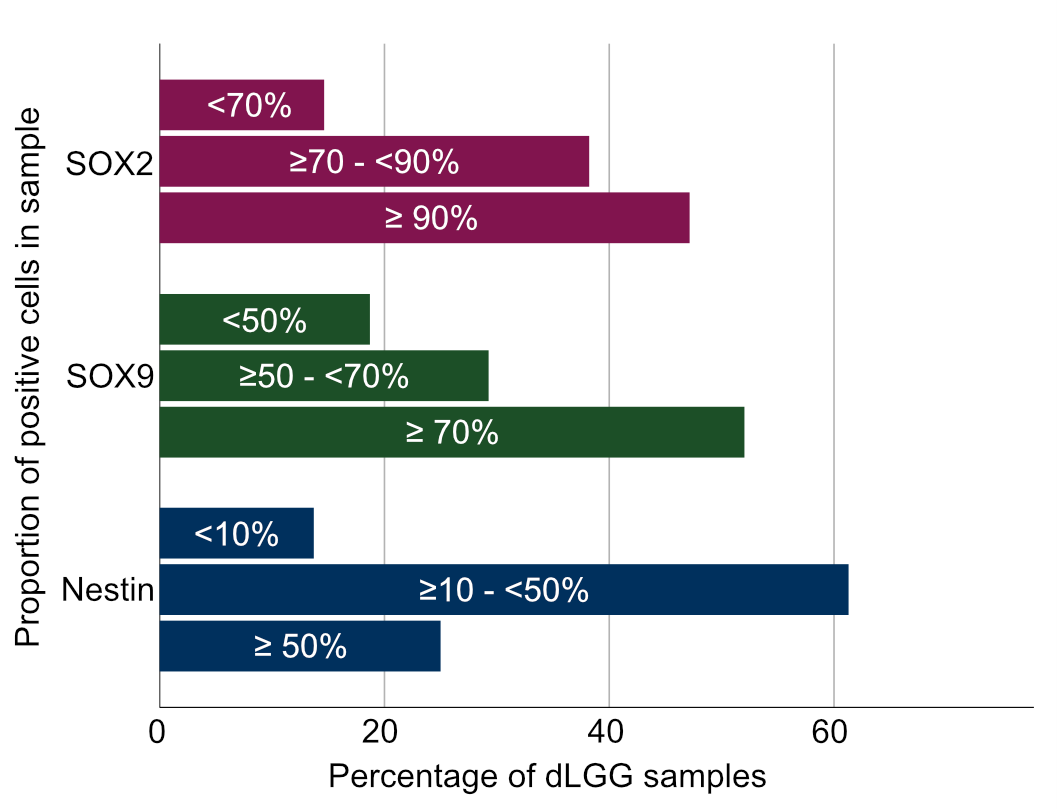

Supplement: vdac074_suppl_Supplementary_Material [file vdac074_suppl_supplementary_material.docx]
